# Perceived Effectiveness and Sustainability of Face Masks Among German Citizens During the 2nd Wave of the COVID-19 Pandemic—A Cross-Sectional Study

**DOI:** 10.3389/fpubh.2022.768454

**Published:** 2022-02-10

**Authors:** Maren Theresa Christin Fendt, Walter Leal Filho, Jelena Barbir, Juliane Boenecke

**Affiliations:** Research and Transfer Centre “Sustainable Development and Climate Change Management” (FTZ-NK), Hamburg University of Applied Sciences, Hamburg, Germany

**Keywords:** masks, COVID-19, Germany, sustainability, effectiveness

## Abstract

**Background:**

The COVID-19 pandemic, which began at the end of 2019, has led to a significant increase in the demand for face masks in Germany and around the globe. Since non-reusable masks are often not correctly disposed of and are not biodegradable, their increasing use harms the environment. Both the ongoing pandemic and the rising environmental pollution eventually pose a threat to human health. Yet, it is unclear whether mask users are conscious of this, and which factors influence their choice of face masks. This study investigates the user preferences, perceived effectiveness, and the sustainability of different mouth/nose protection (MNP) to lay the foundation for developing more sustainable and effective alternatives.

**Methods:**

A national (Germany-wide) cross-sectional study with a sample of 1,036 participants was conducted. Descriptive data analysis was deployed to describe trends and socio-demographic differences among the respondents. Different socio-demographic groups among the respondents were compared regarding their infection risk perception, compliance toward the use of personal protective measures, their choice of MNP, and knowledge level of sustainability and effectiveness of various MNP using inferential statistics (Chi^2^ test/Whitney–Mann-*U*-test/Kruskal–Wallis-test).

**Results:**

The results suggest that, in addition to protective effectiveness, the reusability of MNP is important to not just most respondents but especially to older participants. In contrast, the price, shape, and design were not as important. The knowledge level of the effectiveness and sustainability of MNP was high among the participants and was not associated with socioeconomic characteristics. However, the knowledge level directly influenced the choice of MNP.

**Conclusion:**

There seems to be an inclination to use sustainable MNP, provided their level of protection is similar to medical masks or FFP2/FFP3 masks. The willingness to wear a sustainable option increases with age.

## Introduction

The COVID-19 pandemic has led to an increased global demand for face masks for use by healthcare professionals and the general population as a measure to reduce the viral transmission of SARS-CoV-2. Due to the lack of effective therapeutics and vaccines during the early stage of the pandemic, behavioral measures were, and remain to be, crucial for reducing the risk of infection, such as hand hygiene, coughing/sneezing etiquette, and social distancing. In April 2020, the World Health Organization (WHO) recommended health care professionals and those experiencing symptoms or taking care of sick individuals to use MNP (1).

However, the effectiveness of this measure became the subject of controversial debate worldwide and in Germany, amplified by abrupt changes in mask guidelines and the limited availability of medical masks, which led to confusion and doubt regarding the efficiency of MNPs by the general public (2). Initially, the evidence available was not perceived sufficient by many international and German experts due to the lack and conflicting results of existing clinical trials (3–5). Against this background, experts expressed their concern that the masks could be misused and lead to a false sense of security (6). However, several international observational studies had already pointed to a protective effect of masks (7). During the 2003 SARS outbreak, the frequent use of surgical masks decreased the transmission risk by more than 60% (8). The community-wide use of disposable face masks in Hong Kong, which had the highest risk of COVID-19 importation from China, led to a significantly slower increase in COVID-19 cases at the beginning of the pandemic than in other countries (9). Furthermore, mask use contributed to a decline in influenza cases during the winter, leading to the shortest transmission period in the past 5 years (10). In this context, Cochrane and the World Health Organization (WHO) recommended that the evidence for public health interventions does not necessarily need to be derived from clinical trials (11, 12).

Although the WHO recommendation was shared by several national and international institutions such as the World Medical Association and the Robert Koch Institute (RKI) in Germany, many countries, including Germany, have made masks mandatory for everyone in selected public areas since May 2020 (2, 13). However, to avoid exhausting the supply of professional masks, German citizens were asked to cover their mouth and nose with cotton masks or MNPs instead of disposable masks (14). In June 2020, the WHO officially recommended healthy individuals to wear non-medical masks to secure the supply of medical masks for health care personnel while still controlling the spread of COVID-19 in places where physical distancing was not possible (15).

During the first year of the pandemic, complementary experimental studies showed that cotton, surgical, and N95 (FFP2) masks had a protective effect concerning the transmission of infective droplets (16). In line with these results, the community-wide use of mostly non-medical masks has proven to be effective, as it led to a quantifiable reduction in SARS-CoV-2 transmission in Germany and other countries (7, 9, 17, 18). Besides other infection prevention and control measures, this effect was mainly achieved by the high compliance of the population to wearing MNPs in public (19). Reports from Germany showed that 3% of the population was wearing masks at the beginning of March 2020, increasing to 38% in April and 62% in May. In July 2020, compliance went up to 66% and peaked at 75% at the end of January 2021 (20). A recent systematic review reaffirmed the significant link between the use of face masks and the reduction in transmission of COVID-19 via respiratory droplets (21). Moreover, a recent randomized controlled trail suggests that cloth masks can have a similar protective effect compared to medical masks (22).

While primarily non-medical masks were used in the first half of 2020 (cf. Table 1), the use of non-reusable masks such as medical masks and tightly fitted respirators (filtering face piece, FFP) with filtration efficiencies—characterized as FFP1 (80%), FFP2 (94%), and FFP3 (99%)—increased worldwide with the growing availability. In Germany, disposable masks (mainly medical and FFP2 masks) became increasingly available in 2020, with more than 300 million masks ordered and distributed by the Federal Ministry of Health (29). Toward the end of the second wave, on the 19th of January 2021, medical masks were made compulsory in shopping facilities and public transport, which led to increased compliance and demand (20, 30).

**Table 1:** Effectiveness level, reusability, global use of different masks (before September 2020).

**Mask type**	**Community masks**	**Medical masks**	**FFP1**	**FFP2**	**FFP3**
Filtration efficiency (23)	5–80%	95% (24)	80%	94–95%	99%
Total inwards leakage (25)	60% (26)	35% (26)	22% (27)	8% (27)	2% (27)
Reusability (15)	Recommended	Not recommended	Not recommended	Not recommended	Not recommended
Frequency of use worldwide	72.7%	27.8%		8.4%	0.4% (28)

Even though disposable masks are usually more effective than reusable alternatives (cf. Table 1), their potentially harmful environmental impacts were neglected during the debate related to the pandemic.

Disposable face masks are recommended for single use only and consist of several polymers and fibers (31). Once they are disposed of (littering in public areas, landfills), they start leaking microplastics into the environment as the material polymers break down into smaller pieces (<5 mm) over time (32). Unfortunately, many people are disposing masks incorrectly, contributing to the increasing land and water pollution. According to estimations, approximately 75% of disposable masks and other pandemic-related waste will end up in landfills or the ocean (33). More specifically, about 1.5 million masks or 0.15–0.39 million tons of plastic debris enter global oceans within a year (34). Since disposable face masks take about 450 years to degrade under natural conditions, the adverse effects on the environment are expected to be long-lasting (35).

Given the lasting impacts of plastic debris, it is critical to understand the preferred mask types during the second wave of the COVID-19 pandemic and the factors that influence the choice of a mask while laying the foundation for the development of more sustainable and effective alternatives. Since the influential factors on the selection of masks, such as effectiveness and sustainability, may depend on the individual level of knowledge, another objective was to investigate how well German citizens were informed about the effectiveness and sustainability of different masks. Consequently, it was aimed to analyze how this knowledge was distributed among the German sample and whether it influenced the choice of MNP. The following questions were formulated:

1. Which type of MNP was used the most during the second wave of the pandemic? Which factors influence the choice of MNP among the German sample?
2. How conscious are the participants about the sustainability and effectiveness of different MNP? Is there an association between the level of awareness and the choice of MNP?
3. How are social characteristics (age, education level, perceived risk) related to each of the questions above?

## Materials and Methods

A cross-sectional study design employing an online survey was used to answer the formulated questions. The survey included a total of 20 questions. Before starting the survey, respondents were asked to give their consent (one question). The subsequent 19 questions were divided into four topics: (1) socio-demographic information including country of residence, age group, and educational level (three questions), (2) the perceived risk of getting infected with SARS-CoV-2 and user preferences of MNP in the context of the current COVID-19 pandemic (seven questions), (3) the level of knowledge about the effectiveness, the utility and sustainability of different MNP (eight questions), and (4) the users' attitude toward reusable face masks (one question), which closed the survey. The survey design included single-choice and multiple-choice items, Likert-scale items, and open-ended questions. The questionnaire was available in English and German (Supplementary Material).

The questionnaire's final design was pre-tested using a practice run and feedback interviews with members of the Research and Transfer Centre “Sustainable Development and Climate Change Management”, Hamburg University of Applied Sciences (HAW Hamburg) in Germany and was adjusted for conciseness and clarity. The English and German questionnaires were distributed online via e-mail distribution lists, which were aimed directly at researchers, students of the HAW Hamburg, and partners of the Horizon 2020 project BIO-PLASTICS EUROPE. In addition, the survey was shared via the private and project-related (bioplasticseurope.eu) social media platforms LinkedIn, Facebook, and Instagram for a maximum possible outreach among multiple respondents. Responses were collected for around 4 months (115 days) from October 22, 2020, to February 15, 2021. The survey was repeatedly promoted during this period, primarily at the beginning of November, mid-December 2020, and mid-January 2021. Of the 1,631 international total respondents, 1,050 were living in Germany. Twelve respondents of the German subset were excluded since they did not agree to the consent form. At this stage, 1,038 respondents remained in the sample.

The data was analyzed using descriptive statistics, with frequency description, measures of central tendency, and dispersion. Different socio-demographic sub-groups among the respondents were compared regarding their perceived infection risk, compliance with wearing MNP as a personal protective measure, and their choice of a particular MNP type and aspects of sustainability (cf. Table 2). The group aged 60+, to which only two people were assigned, was not further considered in the analysis due to its small sample size. Consequently, the remaining sample size decreased to 1,036 respondents. For comparison of groups, the Chi^2^ test was used for nominal variables. For 2 × 2 crosstabs and crosstabs containing five or more cells with fewer than five cases, the Fisher's Exact test was applied. In cases of insufficient computing capacity to apply the Fisher's Exact test, the Monte Carlo Simulation was employed. The Mann–Whitney-*U*-test and Kruskal–Wallis test were used for ordinal variables (5-point Likert scale). For Kruskal–Wallis test results indicating a significant difference between groups, pairwise analyses employing the Mann–Whitney-*U*-test with Bonferroni correction were performed. Differences of groups concerning the metric index of knowledge were investigated with the *t*-test and one-way ANOVA after confirming the assumption of a normal distribution. Statistical inference was performed for a significance level of 5%.

**Table 2:** Overview of variable characteristics and statistical tests (binomial, ^†^categorial, ^‡^ordinal).

**Outcome**	**Groups**	**Tests applied**
Perceived infection risk	Education level^‡^	Chi^2^ test
	Age group^‡^	Fisher's Exact test
Compliance with MNP guidelines^‡^	Perceived infection risk	Mann-Whitney-*U*-test
	Education level^‡^	Kruskal-Wallis-test
	Age group^‡^	Kruskal-Wallis-test
Importance of reusability^‡^	Perceived infection risk	Mann-Whitney-*U*-test
	Education level^‡^	Kruskal-Wallis-test
	Age group^‡^	Kruskal-Wallis-test
Most frequent usage of MNP^†^	Perceived infection risk	Chi^2^ test
	Education level^‡^	Fisher's Exact test
	Age group^‡^	Fisher's Exact test
True/false question on modes of protection of MNP^†^	Perceived infection risk	Chi^2^ test
	Education level^‡^	Chi^2^ test
	Age group^‡^	Exact test
	Most frequent usage of MNP^†^	Fisher's Exact test with Monte Carlo Simulation
Perceived self-protection potential of MNP^†^	Perceived infection risk	Chi^2^ test
	Education level^‡^	Fisher's Exact test
	Age group^‡^	Fisher's Exact test
	Most frequent usage of MNP^†^	Fisher's Exact test with Monte Carlo Simulation
Perceived third-party protection potential of MNP^†^	Perceived infection risk	Chi^2^ test
	Education level^‡^	Chi^2^ test
	Age group^‡^	Fisher's Exact test
	Most frequent usage of MNP^†^	Fisher's Exact test with Monte Carlo Simulation
Correct usage cotton mask^†^	Perceived infection risk	Chi^2^ test*
	Education level^‡^	Fisher's Exact test
	Age group^‡^	Fisher's Exact test
	Most frequent usage of MNP^†^	Fisher's Exact test with Monte Carlo Simulation
Correct usage medical mask^†^	Perceived infection risk	Chi^2^ test
	Education level^‡^	Fisher's Exact test
	Age group^‡^	Fisher's Exact test
	Most frequent usage of MNP^†^	Fisher's Exact test with Monte Carlo Simulation
Perceived sustainability of medical masks^†^	Perceived infection risk	Chi^2^ test
	Education level^‡^	Fisher's Exact test
	Age group^‡^	Fisher's Exact test
	Most frequent usage of MNP^†^	Fisher's Exact test with Monte Carlo Simulation
Perceived sustainability of FFP2 mask^†^	Perceived infection risk	Chi^2^ test
	Education level^‡^	Fisher's Exact test
	Age group^‡^	Fisher's Exact test
	Most frequent usage of MNP^†^	Fisher's Exact test with Monte Carlo Simulation
Willingness to choose a biodegradable MNP	Perceived infection risk	Fisher's Exact test
	Education level^‡^	Chi^2^ test
	Age group^‡^	Chi^2^ test
	Most frequent usage of MNP^†^	Chi^2^ test
	Importance of reusability^‡^	Chi^2^ test
Knowledge index on MNP (metric index)	Perceived infection risk	T-test
	Education level^‡^	One-way ANOVA
	Age group^‡^	One-way ANOVA

The online survey was created using the tool Lime Survey. The software RStudio (version 1.4.1103), R (version 4.0.3), SPSS (version 25), and Microsoft Excel (version 2008) were used for data analysis and visualization. The study results were then discussed in the context of the current scientific evidence on MNP effectiveness and aspects of sustainability.

## Results

### Description of the Study Population

A total of 1,036 participants were included in further analysis. The most represented age groups were 18–25 years (58.8%) and 26–35 years (35.3%), whereas the older age groups 36–45 and 46–59 years were less represented, accounting for 4.5 and 1.4%, respectively. Most of the participants had a lower education level, with 47.8% holding a high school degree and 3.6 % holding less than a high school degree. More than one-third of the sample reported having a bachelor's degree (24.6%) or a degree from trade school (16.8%), and they were assigned to the middle education level. The minority belonged to the high education level, with 6.9% of the participants holding a master's degree and 0.3% a PhD or higher. For data analyses, the five education levels were grouped into three categories: “low education level” (51.4%), “middle education level” (41.4%), and “high education level” (7.2%).

**Figure 1:**
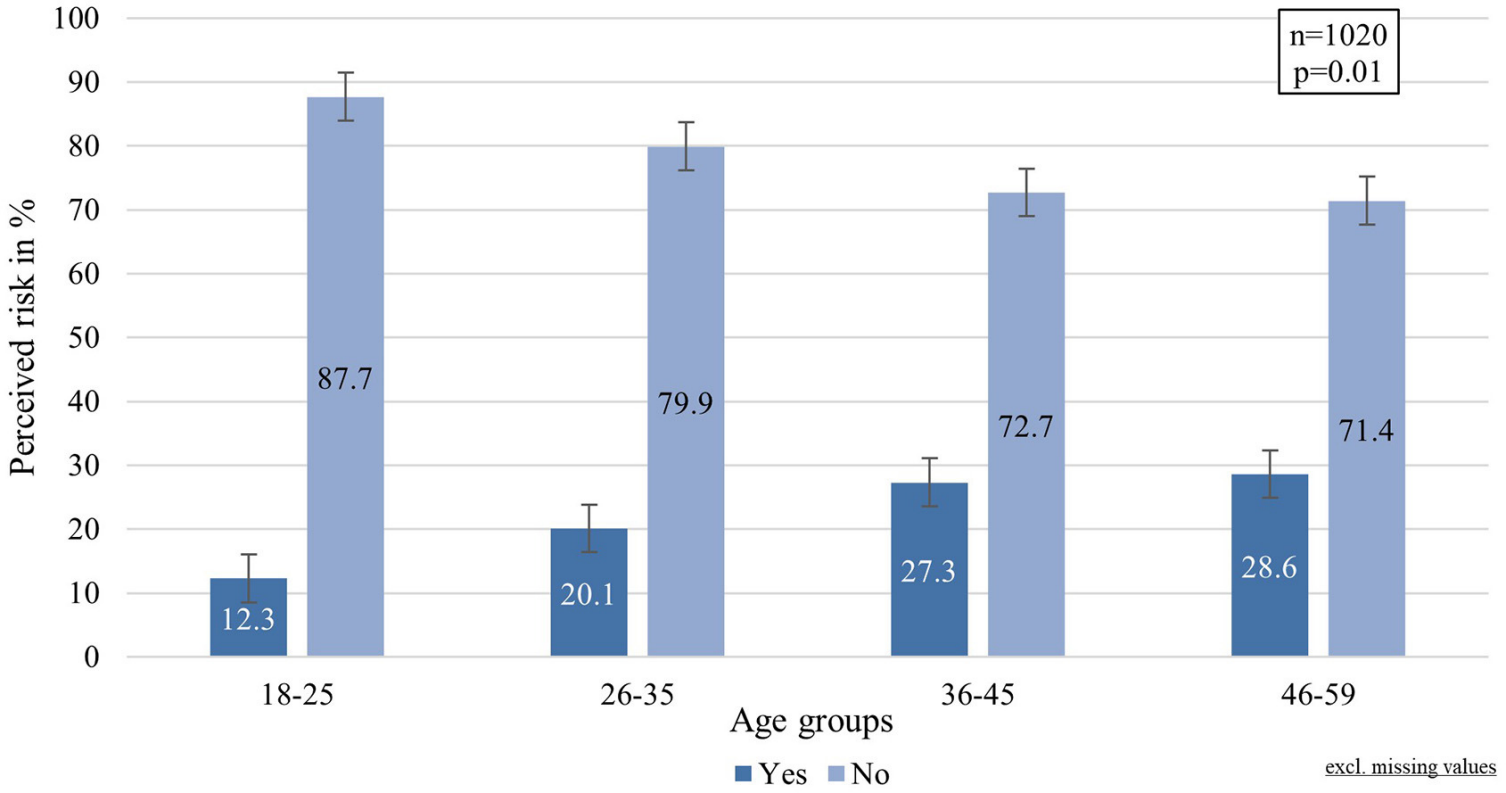
Perceived risk of acquiring SARS-CoV-2 infection by age groups.

The perceived risk of acquiring SARS-CoV-2 infection was assumed to be a factor influencing the choice of MNP (36). At the time of the survey's conduction, most respondents indicated not feeling at an increased risk of getting infected with SARS-CoV-2 (84.1%), whereas 15.9% of the respondents did. The perceived infection risk seemed to increase with age (cf. Figure 1), with the five age groups significantly differing from each other (Chi: 16.624, *p* = 0.01, ϕ:0.127). Besides age, the perceived risk was also associated with the education level, showing a minimal but significant difference between the groups (Chi^2^: 6.772, df:2, *p* = 0.034, φ:0.081).

### Comparison of Groups

#### Aspects of Awareness, Attitude, and Compliance Toward MNP

More than three-quarters of the respondents (81.1%) indicated that they “always” followed the protective guidelines imposed during the pandemic. Almost a third of all participants were “mostly” following the guidelines (16.6%), whereas some (0.9%) were “neutral”. A few were “mostly not” or “never” following the guidelines, with 1.2 and 0.3%, respectively. Further analysis showed that the respondents' compliance significantly differed by age group (H(4): 10.816, *p* = 0.013, η^2^ = 0.007), indicating that the youngest age group (18–25 years) was the most compliant. Pairwise analysis revealed a minimal but significant difference (U:103040.000, *p* = 0.049, *r* = 0.085) between the age groups of 18–25 years (x̄: 4.82) and 26–35 years (x̄: 4.71).

**Figure 2:**
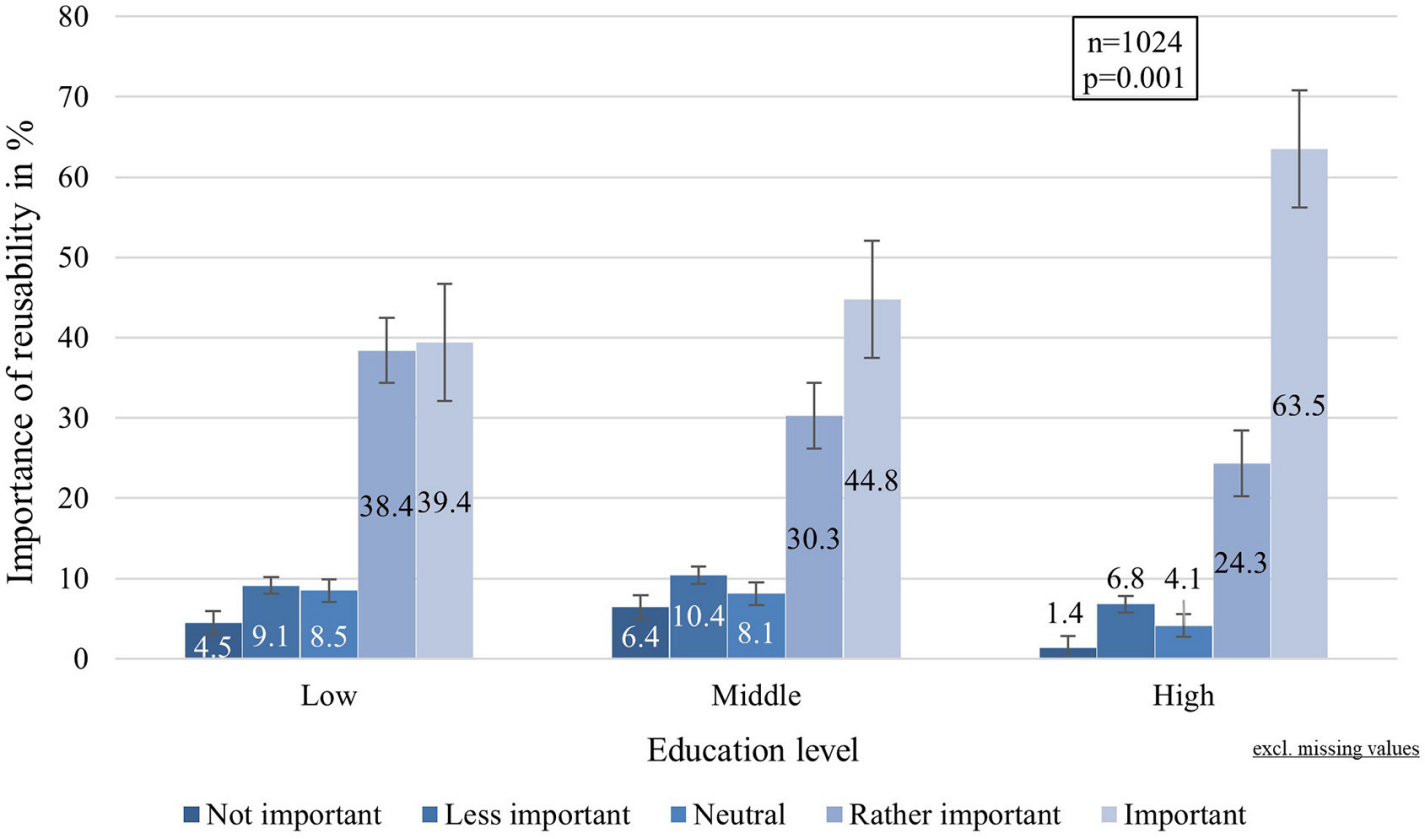
Importance of reusability by education level.

Reusability of MNP was rated “important” by more than half of the participants (43.4%). Approximately one third stated that the reusability of MNP is “rather important” (34.1%), while 8% were “neutral” about the reusability of MNP. A small proportion considered reusability in a MNP “less important” (9.5%) or “not important” (5.1%). Descriptive statistics indicated differences in the distribution between the education groups, especially concerning the answer option of “important” (cf. Figure 2).

Data analysis revealed that the perceived importance of reusability is slightly associated with the education level [H(2): 13.162, df: 2, *p* = 0.001, η^2^ = 0.01]. The pairwise analysis identified a significant difference between the high education level and the middle education level (U:12271, *p* = 0.004, *r* = 0.141) as well as the low education level (U:14675.500, *p* < 0.001, *r* = 0.151), indicating a higher relevance of MNP reusability for the high education group (x̄: 4.42) in comparison to the middle (x̄: 3.97) and low education group (x̄: 3.99).

**Figure 3:**
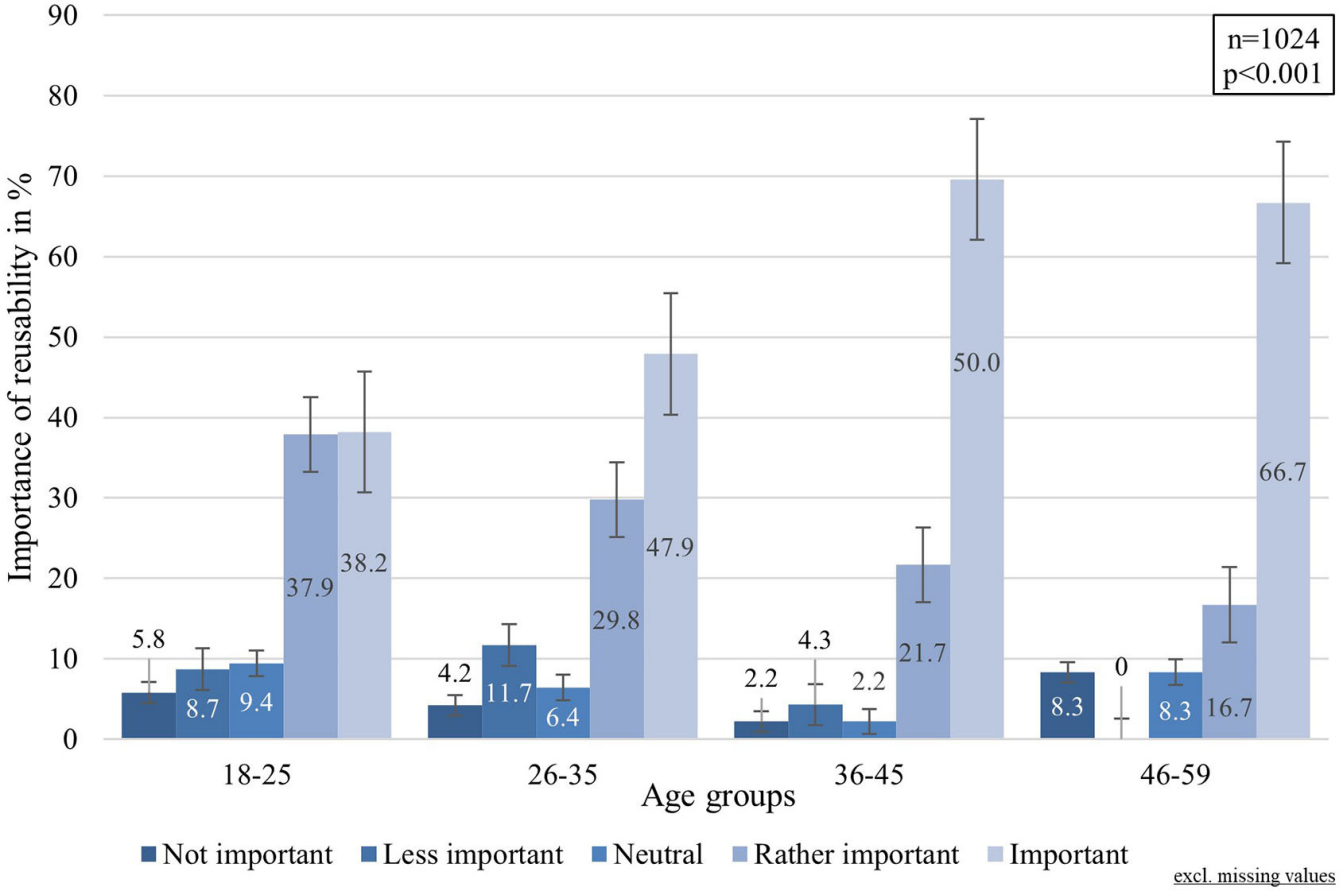
Importance of reusability by age group.

Descriptive statistics further indicated an association between the perceived importance of reusability and age group with regards to the options “rather important“ and “important“ (cf. Figure 3), which was confirmed by the Kruskal-Wallis-test (H(4):20.092, df: 3, *p* < 0.001, η^2^ = 0.016). Pairwise analysis revealed significant differences between the age group of 36–45 (x̄: 4.52), the age group 18–25 (x̄: 3.94) as well as the age group 26–35 (x̄: 4.06), which supports the previous descriptive statistics.

#### MNP Preferences of the Respondents

**Figure 4:**
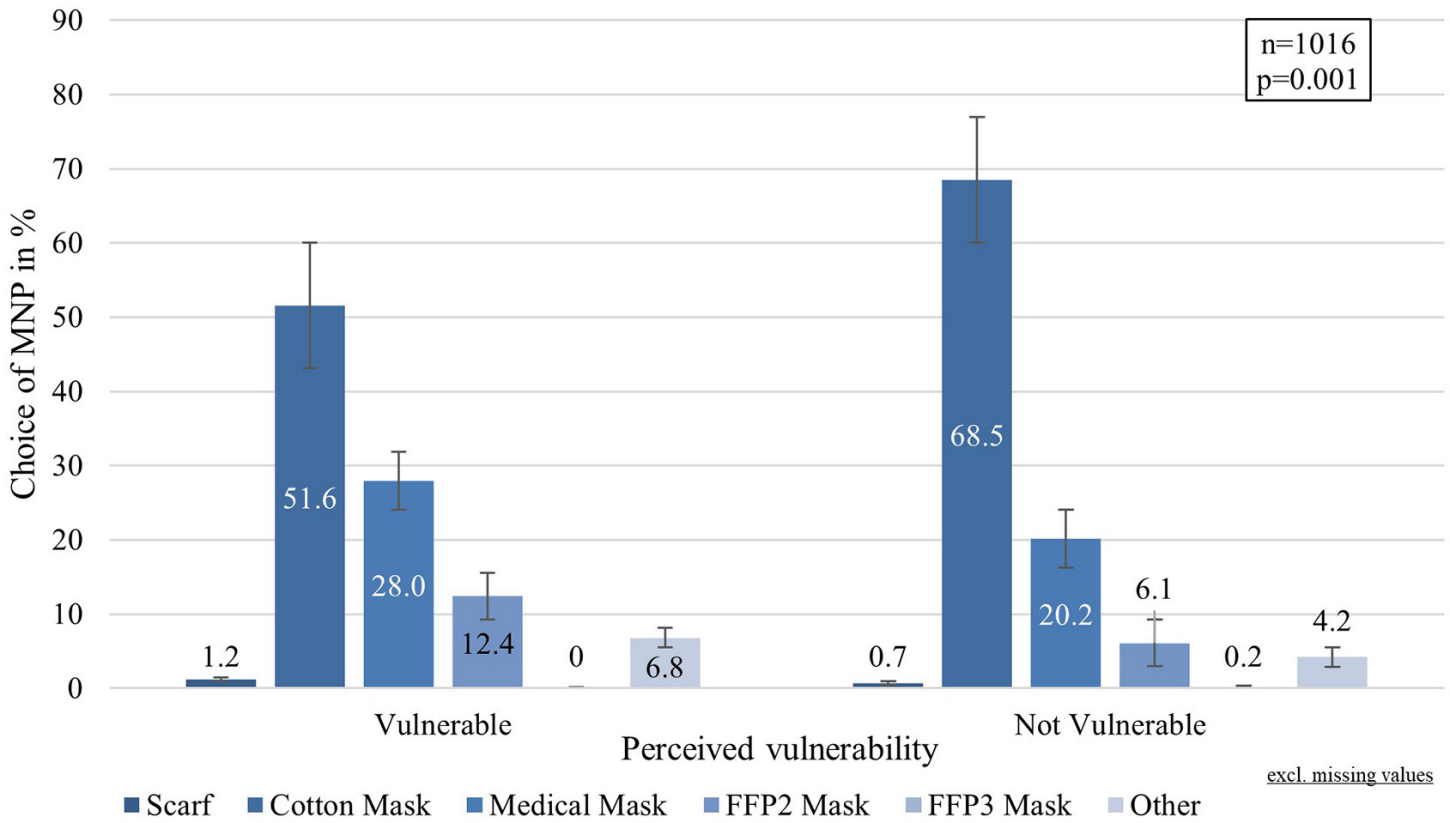
Choice of MNP by perceived risk of infection.

More than half of the respondents (66.0%) reported wearing cotton masks most of the time, followed by 21.2% using medical masks. The more effective FFP2 and FFP3 masks were rarely used, reported by 7.1 and 0.2%, respectively. A scarf was used by 0.8% of the participants, and 4.7% used other options that have not been specified. Further analysis revealed that the choic of MNP was significantly associated with the age group (Chi: 25.270, *p* = 0.042, φ:0.099) and the perceived individual infection risk (Chi^2^: 20.275, df:5, *p* = 0.001, φ:0.141), showing that those who perceive themselves vulnerable wore medical masks and FFP2 masks more often (cf. Figure 4).

When choosing an MNP for daily use, several factors appeared to play a role. The factor “comfort” was considered important by 78.9% of participants, followed by the “protection of others”, which was important to 63.7% when choosing an MNP for daily use. The level of “self-protection“ was important to 54.9%, and more than a third of the participants cared about the MNP's “sustainability level” (46.0%). The “price”, “design”, and “shape” were important to 31.1, 16.3, and 13.2%, respectively. As for which MNP was considered to be the most comfortable, most participants (52.0%) indicated the “cotton mask”, followed by the “medical mask” (34.7%). The other MNPs were rarely indicated as comfortable with FFP2 (4.4%), other (4.3%), scarf (3.8%), and FFP3 (0.6%).

#### Knowledge Level of Different Types of MNP

The knowledge level of the effectiveness level and correct use of different MNP were operationalized by a summed-up metric index of Q1-Q5 during the survey's conduction (cf. Table 3). No differences could be identified between the age groups, education levels and the participants' perceived risk of infection, assuming that all respondents were similarly well-informed about different MNP's protective potential and correct use.

**Table 3:** Questions operationalizing the knowledge level on different types of MNP.

**Q1: Which statement about MNP in everyday application do you think is true?**							**Total *n***
Single choice	Self-protection is given	Third-party protection is given^*^	Both answers are right	Both answers are wrong			
*n* (%)	41 (4.1)	541 (53.6)	231 (22.9)	196 (19.4)			1,009
**Q2: Which type of MNP do you think protects yourself most effectively against SARS-CoV-2 infection in daily life?**							
Single choice	Scarf	FFP2	Cotton Mask	FFP3^*^	Medical mask		
*n* (%)	1 (0.1)	256 (26.6)	18 (1.9)	632 (65.6)	56 (5.8)		963
**Q3: Which type of mouth/nose protection do you think protects others most effectively against Sars-CoV-2 infection in daily life?**							
Single choice	Scarf	FFP2	Cotton Mask	FFP3^*^	Medical mask	All above	
*n* (%)	2 (0.2)	243 (24.8)	29 (3.0)	414 (42.3)	153 (15.6)	137 (14.0)	978
**Q4: How long do you think are you allowed to wear a cotton mask on average to secure its protective function?**							
Single choice	Until it is totally wet	Several weeks (drying in between)	Several days (drying in between)	Only a few hours^*^	<1 h		
*n* (%)	182 (17.9)	17 (1.7)	231 (22.7)	480 (47.1)	109 (10.7)		1,019
**Q5: When do you think a medical mask should be replaced by a new one?**							
Single choice	After one time of wearing^*^	After several times	After several days of wearing	After several weeks of wearing	You never need to exchange it		
*n* (%)	702 (69.7)	247 (24.5)	48 (4.8)	5 (0.5)	5 (0.5)		1,007
**Q6: What is a medical mask made of?**							
Single choice	Cotton	Synthetic polymers (plastics)^*^	Mix of cotton and synthetic polymers	Viscose	Other textiles		
*n* (%)	7 (0.8)	375 (44.0)	434 (50.9)	33 (3.9)	3 (0.4)		852
**Q7: What is a FFP2/FFP3 mask made of?**							
Single choice	Cotton	Synthetic polymers (plastics)^*^	Mix of cotton and synthetic polymers	Viscose	Other textiles		
*n* (%)	1 (0.1)	369 (49.1)	327 (43.5)	43 (5.7)	12 (1.6)		752
**Q8: Which MNP is most likely to be biodegradable? (Multiple Choice)**							
Multiple choice	Cotton mask^*^	Medical mask	Scarf	FFP2 mask	FFP3 mask		
*n* (%)	965 (93.1)	37 (3.6)	124 (12.0)	8 (0.8)	6 (0.6)		1,036

* *Correct answer option*.

The participants' overall knowledge of the effectiveness of different MNP appeared to be high when consulting the descriptive statistics (cf. Table 3). The majority (53.6 %) of the participants described the protective function of different face masks correctly (Q1) by agreeing that all types of face masks provide third-party protection. The Fisher's Exact test revealed a significant association between the outcomes of Q1 and the “Most frequent usage of MNP” (Chi:28.461, *p* = 0.010 φ:0.102).

As for the effectiveness of different MNPs for self-protection (Q2), more than half of the participants (65.6%) chose the correct answer “FFP3”, followed by the “FFP2” (26.6%).

Concerning the MNP's potential to prevent the user from spreading the virus to others (third-party protection), nearly half of the participants (42.3%) chose the “FFP3 mask” correctly to be the most effective MNP (Q4). About a third of the participants chose the wrong option, i.e., “FFP2 mask” (24.8%) or the “cotton mask” (3.0%). Further analysis suggests that knowledge of the third-party protection of different MNP may be associated with the choice of MNP, indicating that the knowledge of the effectiveness of different MNPs influences the preference toward a more effective MNP (Chi:64.919, *p* < 0.001, φ:0.110).

About half (47.1%) of the participants knew about the correct use of a cotton mask (Q4), being aware that this mask type should be exchanged and/or washed after a few hours of wearing to secure its effectiveness. This was significantly related to the choice of MNP (Chi:42.658, *p* < 0.001, φ:0.107) and the perceived infection risk (Chi:9.406, *p* = 0.047, φ:0.098). As for the correct use of a medical mask, 69.9% of the participants knew the right answer (cf. Table 3). With regards to the output of this question (Q5), a significant association toward the choice of MNP could be identified (Chi:59.754, *p* < 0.001, φ:0.123).

Knowledge of the sustainability of different MNPs was sufficient (Q6 and Q7), with 44.0% of the participants choosing the correct material of a medical mask (“synthetic polymers”) and 49.1% answering the same question correctly about FFP2/FFP3 masks (“synthetic polymers”). However, almost half of the participants chose a “mix of cotton and synthetic polymers”, which is wrong for both medical and FFP2/FFP3 masks (50.9 and 43.5%, respectively). Most of the participants knew about the low biodegradability potential of medical masks, FFP2 and FFP3 masks.

**Figure 5:**
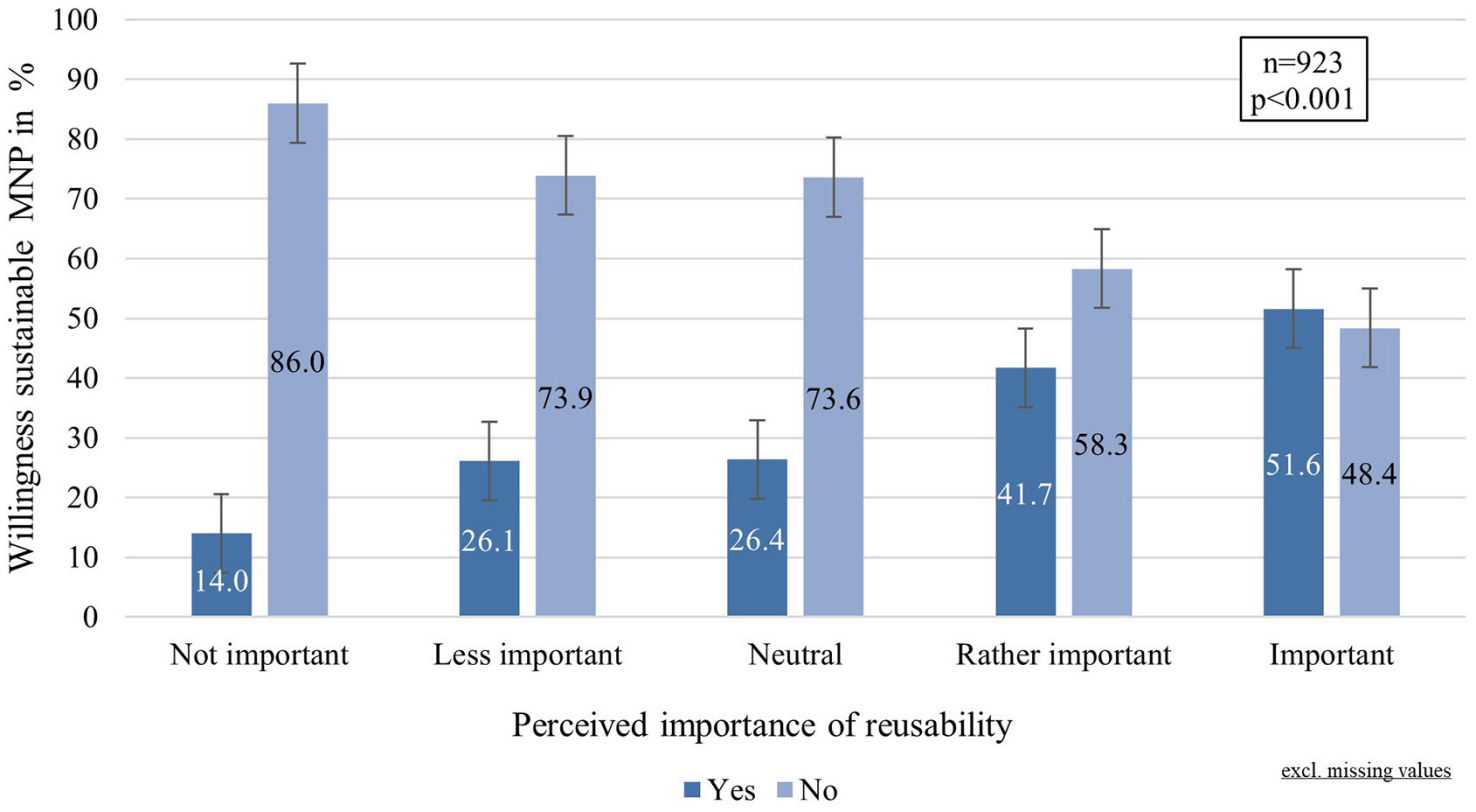
Willingness to choose a sustainable mask by perceived importance of reusability.

A total of 387 participants (41.7%) would choose a biodegradable mask if it provided the same level of protection as a medical mask, even if it were to cost more and looked less fashionable. The majority (58.3%), however, would not choose this sort of mask. The descriptive statistics indicated a relationship between the “willingness to choose a sustainable mask” and the “perceived importance of reusability” (cf. Figure 5), which was confirmed by further analysis, implying a highly significant association and a large effect (Chi^2^:48.016, *p* < 0.001, φ:0.228). The age group (Chi^2^:8.544, df:3, *p* = 0.036, φ:0.096) and the MNP choice (Chi:27.143, *p* < 0.001, φ:0.170) were also significantly related to the “willingness to choose a sustainable mask”.

## Discussion

This cross-sectional study investigated the perceptions and knowledge of 1,036 German participants regarding the effectiveness and sustainability of different MNP associated with their socio-demographic characteristics and perceived SARS-CoV-2 infection risk. The study thereby provides insight into the second wave of the COVID-19 pandemic in Germany, during which the German government had already imposed the use of MNP.

According to the results, the majority of the respondents wore cotton masks daily, followed by medical masks. In contrast, more effective masks, such as FFP2 and FFP3, were rarely used. Furthermore, associations were identified between the MNP used and age group as well as the perceived risk, indicating that older individuals who perceive themselves at increased risk were more likely to use the more effective mask types. Those trends in the usage of different MNPs are confirmed by a similar survey conducted in Germany at the start of the pandemic (37). This national survey showed MNP preference differences between age groups and supports our result that older people use professional instead of homemade masks (37). In the international context, similar usage frequencies were reported in the UK (38), whereas in Asian countries such as China, medical masks and N95 masks were the preferred choice over the same period (39, 40). This difference could stem from higher production capacities in China, which produced half of the number of professional masks needed for the world (41). Other studies indicate that the preferences concerning MNP differed between countries (40, 42), referring to the regulations and communication strategies in place that varied between countries and changed over time (43). In Germany, for example, during the first 3 months of the data collection, it was recommended to wear non-professional MNP, whilst at the end of data collection, medical masks were compulsory in shopping facilities and public transport (44), which has had an impact on the compliance of wearing a mask (20).

The overall compliance toward protective guidelines was high among most study participants, with differences between the age groups. Interestingly, the results show that the youngest age group (18–25 years) was the most compliant with wearing face masks. However, this age group was and continues to be criticized for not following the non-pharmaceutical rules to limit the spread of SARS-CoV-2 (45). While several national publications supported the results of the present study (46, 47), some studies found no association between compliance of wearing masks and age groups (44, 48), and others detected a positive association between increasing age and the likelihood of wearing a mask in public areas (39, 49). The mixed results suggest that the strength of the association varies according to the age group assignment and sample size per age group, which should be considered for the study at hand. In general, some studies indicated the compliance of German citizens toward this measure to be high (49). However, when compared to other European and predominantly Asian countries, the compliance of the German population was relatively low during the second wave (20, 50).

The knowledge about the effectiveness of different masks, on the other hand, seemed high among all participants. Thanks to ongoing research and health education programs, the level of knowledge has increased throughout the pandemic. The majority considered themselves to be well-informed (51), whereas at the beginning of the pandemic young people were insufficiently informed about the effectiveness of masks (46). Most participants knew about different mask types' protective characteristics, without showing any differences between socioeconomic groups. However, an association was found several times between knowledge-related questions (Q1, Q3, Q4, Q5) and the choice of MNP (cf. Table 2), suggesting that knowledge of the effectiveness and sustainability of the MNP could influence their choice of protection.

According to the findings of this study, the users' choice of MNP was primarily influenced by the factors “comfort”, “protection of third parties”, “self-protection”, and “sustainability level”. Similar factors could be identified by other studies, with the most important being “comfort” (42, 52), followed by “efficiency”, “access”, “inconvenience”, and “appearance” (52, 53). In the UK, however, “reusability” was perceived important by most of the people, followed by the “safety” and “comfort”, whilst “price” and “accessibility” were considered less important (38).

Research on the awareness of product sustainability could confirm the positive association of higher education levels and the perceived high importance of reusability in the present study (54–56). For example, one study reported that highly educated people are more likely to behave in an environment-friendly way and reduce, reuse and recycle waste products (56). A similar trend could be observed in the older population when compared to younger age groups (57), supporting our results that older age groups perceive sustainability as more important than younger age groups. Overall, a large proportion of the participants knew about the sustainability level of different types of MNP. Nearly half were open to the use of biodegradable MNP when effective in protection, which differed, however, by age. As previously highlighted, older age groups were more willing to choose a biodegradable and effective but less fashionable mask.

As COVID-19 is known to pose an exceptionally high health risk toward the elderly (58), the imbalanced age distribution in the study population should be considered when interpreting the findings of this study. For example, the overall perceived risk of acquiring SARS-CoV-2 infection was relatively low in the whole study group, which might be due to the increasingly perceived risk of infection that was identified in the older age groups. Similar age-related associations have also been described in other studies (39). The distribution of different age groups among the population of this study may have biased the results, as participants aged 36–45 years accounted for only 4.5% and 46–59 years for only 1.5% of the total sample. The same applies to the education groups (low, medium, high), with the high education group accounting for only 7.2% of the sample. The survey was mainly shared with young students in the middle and lower education groups, given the selected distribution channels such as social media platforms and the HAW Hamburg mailing list. Moreover, the survey was only available online, which may have led to limited outreach to older populations. Finally, the results may not reflect the German population as a whole. Most of the young participants lived in Hamburg and were compliant toward the respective mask-related regulations, which differed from those in other federal states, such as Bavaria, concerning the date of enactment and strictness (59).

It can be concluded that although the knowledge and compliance levels were high among the German participants, the cotton mask was the preferred option during the second wave of the COVID-19 pandemic. The choice of MNP was mainly influenced by the comfort, effectiveness, and sustainability of the mask itself, which implies that in accordance with other outcomes, German citizens—especially older age groups—would be open to a reusable and comfortable solution, which protects themselves and others effectively against SARS-CoV-2.

The results obtained help in determining epidemiological risks identified in the study population and form a basis for further research on more sustainable and effective alternatives of MNP. The current COVID-19 pandemic has already resulted in a large amount of plastic waste that will impact the environment for many years. With these long term consequences in mind, greater emphasis should be placed on the production of more sustainable and environmentally friendly MNPs in epidemic management, especially in view of the growing threat of future epidemics (60).

## Data Availability Statement

The raw data supporting the conclusions of this article will be made available by the authors, without undue reservation.

## Ethics Statement

Ethical review and approval was not required for the study on human participants in accordance with the local legislation and institutional requirements. The participants provided their written informed consent to participate in this study.

## Author Contributions

MTCF: conceptualization, project administration, investigation, methodology, data curation, formal analysis, visualization, writing—original draft, and review and editing—original draft. WL: conceptualization, funding acquisition, supervision, and review and editing—original draft. JBa: conceptualization, methodology, supervision, and review and editing—original draft. JBo: conceptualization, methodology, project administration, supervision, validation, and original draft—review and editing. All authors contributed to the article and approved the submitted version.

## Funding

This study has received funding from the European Union's Horizon 2020 - Research and Innovation Framework Programme through the research project BIO-PLASTICS EUROPE, under grant agreement No. 860407.

## Conflict of Interest

The authors declare that the research was conducted in the absence of any commercial or financial relationships that could be construed as a potential conflict of interest.

## Publisher's Note

All claims expressed in this article are solely those of the authors and do not necessarily represent those of their affiliated organizations, or those of the publisher, the editors and the reviewers. Any product that may be evaluated in this article, or claim that may be made by its manufacturer, is not guaranteed or endorsed by the publisher.
