# Supplementary material for: Perceived Effectiveness and Sustainability of Face Masks Among German Citizens During the 2nd Wave of the COVID-19 Pandemic—A Cross-Sectional Study
Source: Front Public Health. 2022 Feb 10;10:768454. doi: 10.3389/fpubh.2022.768454 (PMC11823475; doi:10.3389/fpubh.2022.768454)
Supplement: Supplementary file 1 [file Data_Sheet_1.PDF]

1 Supplementary Material

Effectiveness and Sustainability of Masks / Mouth and Nose Protections - A Conflict of Interests?

1. Consent

I consent to participate in this survey. I understand my participation is completely voluntary and I can withdraw my consent at any time, without penalty or consequence. I further grant permission for the data generated from this survey to be used in the BIO-PLASTICS EUROPE project activities and scientific publication on the respective topic. I have read and agreed to the privacy policy of Lime Survey (available at https://www.limesurvey.org/en/privacy-policy)*

Choose one of the following answers
Please choose **only one** of the following:

- I agree
- I do not agree

Demographic Data

2. Country of residence *

Please write your answer here:

3. Age group*

Choose one of the following answers
Please choose **only one** of the following:

- 18-25
- 26-35
- 36-45
- 46-59
- 60+

4. Highest degree or level of education *

Choose one of the following answers
Please choose **only one** of the following:

- Less then high school
- High school
- Trade school / Vocational school
- Bachelor’s degree / Interim Diploma (or equivalent)
- Master’s degree / Diploma / State examination (or equivalent)
- PhD or higher

5. Do you consider yourself being especially vulnerable (e.g. risk group, many contacts, increased exposure at work) to aquire a SARS-CoV-2 infection? *

Please choose **only one** of the following:

- Yes
- No
- No Answer

Preferences Of Users

6. To what extend do you follow the guidelines to wear a mouth/nose protection in context of COVID-19?

Choose one of the following answers
Please choose **only one** of the following:

- Always
- Mostly
- Neutral
- Mostly not
- Never

7. How important is it to you that the mouth/nose protection is reusable and/or bio-degradable? *

Choose one of the following answers
Please choose **only one** of the following:

- Important
- Rather important
- Neutral
- Less important
- Not important
- No Answer

8. Which type of mouth/nose protection do you wear most of the time on a daily basis? *

Choose one of the following answers
Please choose **only one** of the following:

- Cotton Mask
- Medical Mask
- Scarf
- FFP2 Mask
- FFP3 Mask
- No Answer
- Other

9. Which mouth/nose protection do you find the most comfortable to wear?

Choose one of the following answers
Please choose **only one** of the following:

- Cotton Mask

- Medical Mask
- Scarf
- FFP2 Mask
- FFP3 Mask
- Other

10. What feature/fact do you consider most important when choosing a mouth/nose protection for daily use? *

Check all that apply
Please choose **all** that apply:

- Comfort
- Sustainability
- Price
- Design
- Selfprotectiveness
- Protection of others
- Shape
- Other:

11. Have you ever had to wear a mouth/nose protection for other reasons than COVID-19 (e.g. working as a health care worker, previous sickness etc.)? *

Choose one of the following answers
Please choose **only one** of the following:

- Yes
- No
- No Answer

Knowlege about the COVID-19 Pandemic and Mouth/Nose Protection

12. Which statement about the mouth/nose protection in everyday application do you think is true?

Choose one of the following answers
Please choose **only one** of the following:

- Protection for others: all types of face masks act as a barrier to effectively trap respiratory secretions so they do not spread to other people or contaminate nearby surfaces
- Protection for yourself: all types of face masks act as an effective transmission barrier to reduce the risk of becoming infected with Sars-CoV-2
- Both answers are right
- Both answers are wrong

13. Which type of mouth/nose protection do you think protects yourself most effectively against Sars-CoV-2 infection in daily life? *

Choose one of the following answers
Please choose **only one** of the following:

- Scarf
- FFP2
- Cotton Mask
- FFP3
- Medical Mask
- No Answer

14. Which type of mouth/nose protection do you think protects others most effectively against Sars-CoV-2 infection in daily life?

Choose one of the following answers
Please choose **only one** of the following:

- Scarf
- FFP2 Mask
- Cotton Mask
- FFP3 Mask
- Medical Mask
- All above

15. How long do you think are you allowed to wear one cotton mask on average (without washing or changing the mask) in order to secure its protective function? *

Choose one of the following answers
Please choose **only one** of the following:

- Until it is totally wet
- Several weeks (when drying it properly in between)
- Several days (when drying it properly in between)
- Only a few hours
- Less than one hour
- No Answer

16. When do you think should a medical mask be replaced by a new one?

Choose one of the following answers
Please choose **only one** of the following:

- After one time of wearing
- After several times of wearing
- After several days of wearing
- After several weeks of wearing
- You never need to exchange it

17. What is a medical mask made of?

Choose one of the following answers
Please choose **only one** of the following:

- Cotton
- Synthetic Polymers (plastics)
- Mix of cotton and synthetic polymers
- Viscose
- Other Textiles

18. What is a FFP2/FFP3 mask made of?

Choose one of the following answers
Please choose **only one** of the following:

- Cotton
- Synthetic Polymers (plastics)
- Mix of cotton and synthetic polymers
- Viscose
- Other textiles

19. Which mouth/nose protection is most likely to be bio-degradable?

Check all that apply
Please choose **all** that apply:

- Cotton Mask
- Medical Mask
- Scarf
- FFP2 Mask
- FFP3 Mask

20. Final Question

Would you choose a bio-degradable mouth/nose protection if it promises the same level of effectiveness as a medical mask but costs more and looks less fashionable?

https://www.littlepinkmaker.com/reusable-masks*

Choose one of the following answers
Please choose **only one** of the following:

- Yes
- No
- No Answer

Effektivität und Nachhaltigkeit von Masken / Mund-Nasen-Schutz - Ein Interessenskonflikt?

1. Einverständniserklärung

Ich stimme der Teilnahme an dieser Umfrage zu. Ich verstehe, dass meine Teilnahme völlig freiwillig ist und ich meine Einwilligung jederzeit konsequenzlos widerrufen kann. Ferner gestatte ich, dass die aus dieser Erhebung gewonnenen Daten für die Projektaktivitäten von BIO-PLASTICS EUROPE und die wissenschaftliche Veröffentlichung zum jeweiligen Thema verwendet werden dürfen. Ich habe die Datenschutzerklärung von Lime Survey (verfügbar unter https://www.limesurvey.org/en/privacy-policy) gelesen und damit einverstanden.*

Bitte wählen Sie eine der folgenden Antworten:
Bitte wählen Sie nur eine der folgenden Antworten aus:

- Ich stimme zu
- Ich stimme nicht zu

Demografische Daten

2. Bitte benennen Sie das Land in dem sich Ihr dauerhafter Wohnsitz befindet*

Bitte geben Sie Ihre Antwort hier ein:

3. Bitte wählen Sie Ihre Altersgruppe*

Bitte wählen Sie eine der folgenden Antworten:
Bitte wählen Sie nur eine der folgenden Antworten aus:

- 18-25
- 26-35
- 36-45
- 46-59
- 60+

4. Bitte benennen Sie Ihren höchsten Bildungsabschluss*

Bitte wählen Sie eine der folgenden Antworten:
Bitte wählen Sie nur eine der folgenden Antworten aus:

- Realschulabschluss / Fachgebundene Hochschulreife
- Allgemeine Hochschulreife
- Abgeschlossene Berufsausbildung
- Abgeschlossenes Bachelorstudium / Zwischendiplom (oder gleichwertig)
- Abgeschlossenes Masterstudium / Diplom / Staatsexamen (oder gleichwertig)
- Promotion (Doktor) oder höherwertig

5. Empfinden Sie sich selbst als besonders gefährdet, (z.B. Risikogruppe, viele Personenkontakte, erhöhtes Risiko bei der Arbeit) an COVID-19 zu erkranken? *

Bitte wählen Sie eine der folgenden Antworten:
Bitte wählen Sie nur eine der folgenden Antworten aus:

- Ja
- Nein
- Keine Antwort

Nutzerpräferenzen

6. Inwieweit folgen Sie den Richtlinien, eine Mund-Nasen-Schutz in Zeiten der COVID-19-Pandemie zu tragen?

Bitte wählen Sie eine der folgenden Antworten:
Bitte wählen Sie nur eine der folgenden Antworten aus:

- Immer
- Meistens
- Weder noch
- Meistens nicht
- Nie

7. Wie wichtig ist es Ihnen, dass der Mund-Nasen-Schutz wiederverwendbar und/oder biologisch abbaubar ist? *

Bitte wählen Sie eine der folgenden Antworten:
Bitte wählen Sie nur eine der folgenden Antworten aus:

- Wichtig
- Eher wichtig
- Weder noch
- Weniger wichtig
- Nicht wichtig
- Keine Antwort

8. Welche Art von Mund-Nasen-Schutz tragen Sie täglich die meiste Zeit? *

Bitte wählen Sie eine der folgenden Antworten:
Bitte wählen Sie nur eine der folgenden Antworten aus:

- Baumwoll-Maske
- Medizinische Maske
- Schal
- FFP2 Maske
- FFP3 Maske
- Keine Antwort
- Sonstiges

9. Welchen Mund-Nasen-Schutz finden Sie am bequemsten zu tragen?

Bitte wählen Sie eine der folgenden Antworten:
Bitte wählen Sie nur eine der folgenden Antworten aus:

- Baumwoll-Maske
- Medizinische Maske
- Schal
- FFP2 Maske
- FFP3 Maske
- Sonstiges

10. Welche Eigenschaft halten Sie bei der Wahl eines Mund-Nasen-Schutzes für den täglichen Gebrauch besonders wichtig? *

Bitte wählen Sie die zutreffenden Antworten aus:
Bitte wählen Sie alle zutreffenden Antworten aus:

- Komfort
- Nachhaltigkeit
- Preis
- Design
- Selbstschutz
- Fremdschutz
- Form
- Sonstiges

11. Mussten Sie schon einmal aus anderen Gründen als der COVID-19-Pandemie einen Mund-Nasen-Schutz tragen (z.B. Arbeit im Gesundheitswesen, aufgrund einer Grunderkrankung.)? *

Bitte wählen Sie eine der folgenden Antworten:
Bitte wählen Sie nur eine der folgenden Antworten aus:

- Ja
- Nein
- Keine Antwort

Wissensstand zur COVID-19-Pandemie und Mund-Nasen-Schutz

Welche Aussage über den Mund-Nasen-Schutz in der täglichen Anwendung sind Ihrer Meinung nach wahr?

Bitte wählen Sie eine der folgenden Antworten:
Bitte wählen Sie nur eine der folgenden Antworten aus:

- Fremdschutz: alle Arten von Gesichtsmasken sind geeignet, um größere ausgeatmete Tröpfchen effektiv abzufangen und somit einer Kontamination von Personen oder nahe gelegenen Oberflächen vorzubeugen
- Eigenschutz: alle Arten von Gesichtsmasken wirken als Übertragungsbarriere und reduzieren das Risiko, sich mit Sars-CoV-2 zu infizieren
- Beide Antworten sind richtig
- Beide Antworten sind falsch

Welche Art von Mund-Nasen-Schutz schützt Sie Ihrer Meinung nach am effektivsten vor einer Sars-CoV-2-Infektion im Alltag? *

Bitte wählen Sie eine der folgenden Antworten:
Bitte wählen Sie nur eine der folgenden Antworten aus:

- Schal
- FFP2 Maske
- Baumwoll-Maske
- FFP3 Maske
- Medizinische Maske
- Keine Antwort

Welche Art von Mund-Nasen-Schutz schützt Ihrer Meinung nach andere am effektivsten vor einer Sars-CoV-2-Infektion im Alltag?

Bitte wählen Sie eine der folgenden Antworten:
Bitte wählen Sie nur eine der folgenden Antworten aus:

- Schal
- FFP2 Maske
- Baumwoll-Maske
- FFP3 Maske
- Medizinische Maske
- Alle Antwortmöglichkeiten

Wie lange dürfen Sie ihrer Meinung nach im Durchschnitt eine Baumwollmaske tragen (ohne Waschen oder Wechseln der Maske) ohne zu riskieren, dass ihre Barrierefunktion nachlässt? *

Bitte wählen Sie eine der folgenden Antworten:
Bitte wählen Sie nur eine der folgenden Antworten aus:

- Bis sie durchnässt ist
- Mehrere Wochen (wenn sie zwischen den Tragezeiten gut durchtrocknet)
- Mehrere Tage (wenn sie zwischen den Tragezeiten gut durchtrocknet)
- Ein paar Stunden
- Weniger als eine Stunde
- Keine Antwort

Wann sollten Sie ihrer Meinung nach eine medizinische Maske durch eine neue ersetzen?

Bitte wählen Sie eine der folgenden Antworten:
Bitte wählen Sie nur eine der folgenden Antworten aus:

- Nach einem Mal tragen
- Nach mehreren Malen des Tragens
- Nach mehreren Tagen des Tragens
- Nach mehreren Wochen des Tragens
- Sie muss nie gewechselt werden

Aus welchem Material besteht eine medizinische Maske?

Bitte wählen Sie eine der folgenden Antworten:
Bitte wählen Sie nur eine der folgenden Antworten aus:

- Baumwolle
- Synthetischen Polymeren (Kunststoffe)
- Gemisch aus Baumwolle und synthetischen Polymeren
- Viskose
- Anderen Textilien

Aus welchem Material bestehen FFP2/FFP3 Masken?

Bitte wählen Sie eine der folgenden Antworten:
Bitte wählen Sie nur eine der folgenden Antworten aus:

- Baumwolle
- Synthetische Polymeren (Kunststoff)

- Einem Gemisch aus Baumwolle und synthetischen Polymeren
- Viskose
- Anderen Textilien

Welcher Mund-Nasen-Schutz ist am ehesten biologisch abbaubar?

Bitte wählen Sie die zutreffenden Antworten aus:
Bitte wählen Sie alle zutreffenden Antworten aus:

- Baumwoll-Maske
- Medizinische Maske
- Schal
- FFP2 Maske
- FFP3 Maske

Abschließende Frage

Würden Sie einen biologisch abbaubaren Mund-Nasen-Schutz wählen, wenn sie das gleiche Maß an Wirksamkeit verspricht wie eine medizinische Maske, aber mehr kostet und weniger modisch aussieht (siehe unten)?

https://www.littlepinkmaker.com/reusable-masks*

Bitte wählen Sie eine der folgenden Antworten:
Bitte wählen Sie nur eine der folgenden Antworten aus:

- Ja
- Nein
- Keine Antwort
